# Serum imbalance between the extracellular matrix metalloproteinases (MMP-2 and MMP-9) and their tissue inhibitor (TIMP-1) in patients with food and airborne allergy

**DOI:** 10.1186/2045-7022-3-S3-P50

**Published:** 2013-07-25

**Authors:** A Kuźmiński, M Przybyszewski, M Graczyk, M Żbikowska-Gotz, Z Bartuzi

**Affiliations:** 1 Department of Allergology, Clinical Immunology and Internal Medicine, Collegium Medicum in Bydgoszcz, Nicolaus Copernicus University in Toruń, Bydgoszcz, Poland

## Background

Comparison of the concentration of MMP-2 and MMP-9 and TIMP-1 in patients with food and airborne allergy as compared to patients without allergy.

## Methods

The study was performed in 80 individuals: 60 patients with exacerbation of allergic disease (30 with food allergy and 30 with airborne allergies) and 20 healthy subjects. We examined the serum concentrations of soluble forms of MMP-2, MMP-9 and TIMP-1. Determination of these parameters was performed by ELISA. For MMP-9 and TIMP-1 was used kit from Bender MedSystems, for MMP-2 of RayBiotech. A statistical study results was performed using the computer program STATISTICA 9.1.

## Results

The concentrations of sMMP-2 and-9 in the groups of patients with food and airborne allergy and control groups were, respectively, 153,8±97,1 and 198,8±51,4 ng/ml; 136,3±41,2 and 184,9±38,8 ng/ml, and 119,5±12,5 and 121,6±25,5 ng/ml. sMMP-2 demonstrated statistically significant differences between the group with food allergies and the control group (p = 0.0309), no significant differences between the group of airborne allergy and the control group, as well as between groups of airborne and food allergies (for p = 0.4225 and p = 0.1473). Differences of sMMP-9 levels were significantly higher in the group of airborne and food allergies than in the control group (both P = 0.0000). There was no significant difference between the group of patients with airborne and food allergy (p = 0.3952). The concentrations of sTIMP-1 in groups of patients with food allergy and airborne were significantly higher than those in the control group (respectively p = 0.0000 and p = 0.0003) and were in the group with food allergies 164.3 ± 59.2 ng/ml; airborne allergy ± 145.4 50.1 ng/ml, whereas in the control group 92.4 ± 26.7 ng/ml. There was no statistically significant difference sTIMP-1 concentrations between the group of patients with airborne and food allergy (p = 0.2458).

## Conclusion

MMP-2 and MMP-9 and TIMP-1 were significantly higher in patients with food allergy than in the control group. A similar observation (except for concentrations sMMP-2) also applies to a group of patients with airborne allergy. The results of this study suggest an important role of MMPs and TIMPs in the pathogenesis of allergic inflammation.

## Disclosure of interest

None declared.

